# Spatial Representations in the Human Brain

**DOI:** 10.3389/fnhum.2018.00297

**Published:** 2018-07-30

**Authors:** Nora A. Herweg, Michael J. Kahana

**Affiliations:** Computational Memory Lab, Department of Psychology, University of Pennsylvania, Philadelphia, PA, United States

**Keywords:** spatial memory, navigation, cognitive map, episodic memory, MTL, theta, place cells, grid cells

## Abstract

While extensive research on the neurophysiology of spatial memory has been carried out in rodents, memory research in humans had traditionally focused on more abstract, language-based tasks. Recent studies have begun to address this gap using virtual navigation tasks in combination with electrophysiological recordings in humans. These studies suggest that the human medial temporal lobe (MTL) is equipped with a population of place and grid cells similar to that previously observed in the rodent brain. Furthermore, theta oscillations have been linked to spatial navigation and, more specifically, to the encoding and retrieval of spatial information. While some studies suggest a single navigational theta rhythm which is of lower frequency in humans than rodents, other studies advocate for the existence of two functionally distinct delta–theta frequency bands involved in both spatial and episodic memory. Despite the general consensus between rodent and human electrophysiology, behavioral work in humans does not unequivocally support the use of a metric Euclidean map for navigation. Formal models of navigational behavior, which specifically consider the spatial scale of the environment and complementary learning mechanisms, may help to better understand different navigational strategies and their neurophysiological mechanisms. Finally, the functional overlap of spatial and declarative memory in the MTL calls for a unified theory of MTL function. Such a theory will critically rely upon linking task-related phenomena at multiple temporal and spatial scales. Understanding how single cell responses relate to ongoing theta oscillations during both the encoding and retrieval of spatial and non-spatial associations appears to be key toward developing a more mechanistic understanding of memory processes in the MTL.

## Introduction

Space is one of the most fundamental dimensions along which we organize our perceptions and memories. Kant wrote in 1781 *“Space is a necessary a priori representation that underlies all outer intuitions (Der Raum ist eine notwendige Vorstellung a priori, die allen äußeren Anschauungen zum Grunde liegt)"* (Kant, 1781). Every *outer intuition*, every object we perceive or imagine possesses a specific shape and occupies a specific location in space. Memory for spatial relations and spatial contexts associated with specific experiences thereby helps us navigate and interact with the objects we encounter. There is an extensive body of research on the neurophysiology of spatial memory in rodents, in which invasive brain recordings can be collected during navigation. Memory research in humans, in turn, had traditionally focused on verbal memory tasks without a spatial component. Here we review studies that have begun to address this gap using virtual navigation tasks in combination with electrophysiological recordings and neuroimaging in humans. These studies are the basis for our understanding of inter-species differences and similarities in spatial memory and further inform the ongoing debate on the generality or specificity of coding spatial vs. conceptual information (Eichenbaum et al., 1999; Milivojevic and Doeller, 2013; Constantinescu et al., 2016).

Spatial coordinates can be extracted from various sensory inputs including visual (Goodale and Milner, 1992; Kravitz et al., 2011), auditory (King et al., 2011; Salminen et al., 2012) and somatosensory (Badde and Heed, 2016) signals. Based on studies in humans (Silver and Kastner, 2009; Galati et al., 2010) and non-human primates (Andersen et al., 1997), there is strong consensus that these signals are integrated in the posterior parietal cortex (PPC). Here, they are combined with proprioceptive information and translated between *egocentric* reference frames centered on different body-parts (e.g., eye, head, or hand) to facilitate movement planning with different effectors (Grefkes and Fink, 2005; Medendorp et al., 2005; Moon et al., 2007; Van Der Werf et al., 2008; Filimon, 2010; Herweg et al., 2014). Egocentric coordinates can be distinguished from *allocentric* (Burgess, 2006) coordinates (sometimes referred to as world-centered or object-centered), which represent spatial information with respect to external reference frames (e.g., with respect to specific objects or along salient dimensions of an environment) and, hence, are independent of the position of the individual. Allocentric spatial coding has mainly been associated with the medial temporal lobe (MTL) and forms the basis for a *cognitive map* (Tolman, 1948) of the environment, from which information on the spatial relations of landmarks or objects can be flexibly extracted when needed. First described by Tolman (1948), the idea of a cognitive map pioneered model-based learning in general (Doll et al., 2012) and inspired research on the neural basis of an allocentric spatial map in particular.

In the following sections, we will review neurophysiological evidence for an allocentric spatial map in the human MTL, which is used to code observer-independent spatial relations during (virtual) navigation, exploration, or imagination (see section “An Allocentric Spatial Map in the Human MTL?”). We then consider interactions between this spatial map in the MTL and other brain regions involved in the encoding and retrieval of spatial information, such as the PPC and prefrontal cortex (PFC; see section “Spatial Representations in a Brain-Wide Network”). Although the idea of a spatial map is tightly linked to spatial navigation, behavioral studies on human way finding suggest complementary learning and decision processes, which we will highlight in Section “Cognitive Mapping and Complementary Learning Mechanisms in Human Spatial Navigation.” Finally, we will consider the role of the MTL in declarative memory formation and retrieval more broadly (see section “Functional Overlap in the MTL: A Common Map for Physical and Conceptual Space?”) and propose avenues for future research.

## An Allocentric Spatial Map in the Human MTL?

Studies of awake behaving rodents claim to have identified the building blocks of an allocentric spatial map in the firing of individual neurons and neuronal oscillations in the MTL (Moser et al., 2008). This section reviews recent progress in translating these findings to human navigation.

### Spatially Selective Single Cells – Observed via Invasive Recordings or Inferred From Population Activity

Hippocampal place cells (O’Keefe, 1976, 1979) increase their firing rate whenever an animal traverses a particular place in the environment (O’Keefe and Conway, 1978) and entorhinal grid cells preferentially fire on the vertices of a hexagonal grid (Hafting et al., 2005). These cells represent an animal’s spatial location with respect to landmarks and spatial boundaries, and often independent of the animal’s facing direction. We and others (Moser et al., 2008), therefore interpret these findings to be consistent with an allocentric reference frame (although alternative accounts exist: Wolbers and Wiener, 2014; Filimon, 2015). While early fMRI studies confirm a role for the human MTL in spatial navigation (Aguirre et al., 1996; Maguire et al., 1998; Bohbot et al., 2004; Wolbers et al., 2007; for a review see Maguire et al., 1999), more direct insight at the cellular level comes from intracranial recordings in patients with pharmaco-resistant epilepsy. In these patients, micro-wire bundles extending from the tip of medial temporal depth electrodes (Misra et al., 2014) can be used to record single unit spiking activity.

**FIGURE 1:**
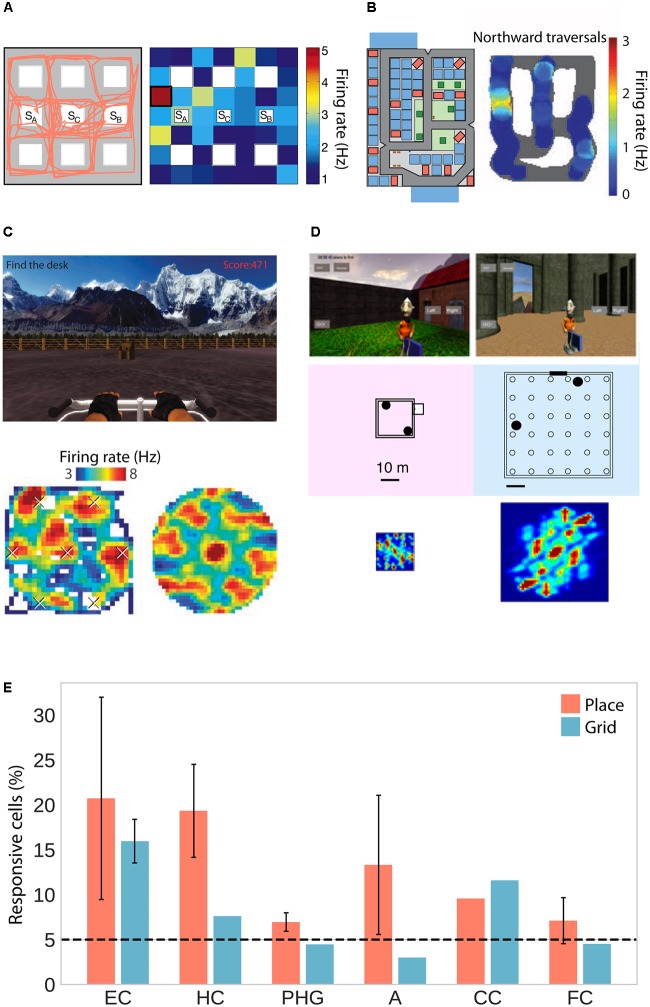
Place and grid cell activity in humans. **(A)** Traveled path *(left)* and firing rate map of an exemplary place cell (*right*) in a city block environment. Adapted with permission (Ekstrom et al., 2003) from Springer Nature. **(B)** City environment (*left*) and firing rate map for an exemplary unidirectional place cell firing only during northward traversals (*right*). Adapted with permission (Miller et al., 2013) from Science. **(C)** Open-field environment (*top*), firing rate map (*lower left*), and spatial autocorrelation function (*lower right*) of an exemplary grid cell. Adapted with permission (Jacobs et al., 2013) from Springer Nature. **(D)** Small (*left*) and large (*right*) virtual arena with corresponding autocorrelation functions showing small and large grid spacing, respectively. Adapted with permission (Nadasdy et al., 2017) from PNAS. **(E)** Percentage of place- and grid-responsive cells in the entorhinal cortex (EC), hippocampus (HC), parahippocampal gyrus (PHG), amygdala (A), cingulate cortex (CC), and frontal cortex (FC). Data were summarized from Ekstrom et al. (2003); Jacobs et al. (2013), Miller et al. (2013), and Nadasdy et al. (2017). Standard error of the mean is shown if more than one of the studies reported data for a given brain region.

As human patients navigated virtual environments, place-selective cells were observed in three independent studies in the hippocampus, parahippocampal gyrus (including entorhinal cortex) and amygdala (**Figures 1A,B,E**; Ekstrom et al., 2003; Jacobs et al., 2013; Miller et al., 2013). One of these studies only considered cells whose firing rate was a function of place but not view, and found those cells to be significantly clustered in the hippocampus with an average of 1.7 non-contiguous place-fields (Ekstrom et al., 2003). Moreover, two studies found predominantly omnidirectional coding (i.e., same firing rate for different directions; Ekstrom et al., 2003; Jacobs et al., 2013) and one study found predominantly unidirectional coding (Miller et al., 2013). In rodents, omnidirectional and unidirectional place cells are associated with open field and maze-like environments, respectively (McNaughton et al., 1983; Muller et al., 1994), suggesting that traversal of an area in different directions allows for omnidirectional representations, which are independent of one particular serial order of processing (Buzsáki, 2005). The data on human place-responsive cells aligns with this idea – while Jacobs et al. (2013) used an open field environment, the environment used in Miller et al. (2013) featured constrained paths and high buildings. Although Ekstrom et al. (2003) also used a city environment, the paths were wider and the buildings smaller than in the study by Miller and colleagues, allowing for higher variability in taken paths and visibility of large portions of the environment from any one location.

In addition to these putative place cells, studies in humans have also confirmed the existence of grid cells, which fire at the vertices of a hexagonal grid spanning the environment. Two studies observed grid-like firing with sixfold rotational symmetry in the human entorhinal cortex and hippocampus in a virtual open-field environment (**Figures 1C–E**; Jacobs et al., 2013; Nadasdy et al., 2017). Moreover, navigation in a circular path environment elicited spatially periodic activity without rotational symmetry in the entorhinal cortex, which may reflect grid cell activity anchored to individual corridors or some other form of distance coding (Miller et al., 2015). One of the studies showing sixfold rotational symmetry estimated grid spacing (i.e., the distance between adjacent grid nodes) to be at least 1–6 m in the real world (Jacobs et al., 2013). The other study showed that spacing was a function of environment size, with coarser spacing in larger environments (**Figure 1D**; Nadasdy et al., 2017). Studies in rats suggest that neighboring (Hafting et al., 2005) and distant (Barry et al., 2007) grid cells share a similar orientation. In a small sample of epilepsy patients, orientation was consistent across patients and anchored to environmental geometry (i.e., square vs. rectangular shaped; Nadasdy et al., 2017).

**FIGURE 2:**
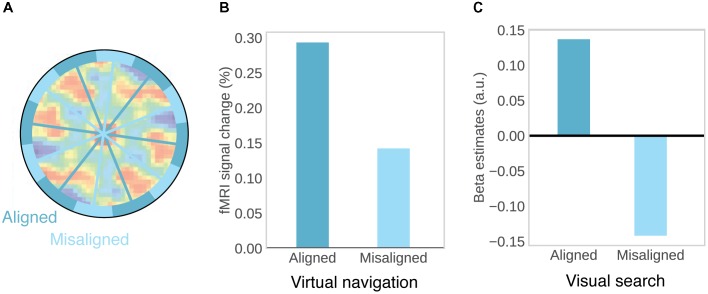
Evidence for grid-cells in fMRI BOLD. **(A)** Rationale for observing grid-like activity in BOLD contrast. Based on the assumption that grid cells show a similar grid orientation across the population, movement in a direction aligned with the common grid direction should be associated with higher firing rates across the population and therefore higher BOLD signal than movement in a direction misaligned with the grid. Underlying autocorrelation function adapted with permission (Jacobs et al., 2013) from Springer Nature. **(B)** During virtual navigation, movement in a direction aligned with grid orientation, which was estimated from an independent subset of the data, resulted in higher BOLD in the entorhinal cortex than movement in a direction misaligned with the grid. Data from Doeller et al. (2010). **(C)** Analogously, eye movements in a direction aligned with grid orientation in a visual tracking task are associated with higher BOLD than eye movements in a direction misaligned with the grid. Data from Nau et al. (2018).

Consistent orientation across cells is the basis for fMRI studies on grid-like activity, as movements in a direction aligned with the common grid direction should be associated with higher firing rates, and increased BOLD signal, than movements in a direction unaligned with the grid (**Figure 2A**). This effect may be further increased by conjunctive grid × head direction cells which fire at the vertices of the grid only at one particular running direction (Doeller et al., 2010). The BOLD contrast for aligned vs. misaligned trajectories can be calculated after initially estimating grid orientation on a subset of the data. Using this logic, grid-like activity in BOLD signal was observed in the entorhinal cortex (**Figure 2B**; Doeller et al., 2010; Kunz et al., 2015; Stangl et al., 2018). Separate studies observed the same effect during imagined movement (Horner et al., 2016) and stationary heading (Bellmund et al., 2016). Furthermore, grid orientation varied across subjects, ruling out that visual features of the environment were driving the effects (Doeller et al., 2010; Horner et al., 2016) and the coherence of grid orientations within each subject was correlated with spatial memory performance (Doeller et al., 2010; Kunz et al., 2015). In agreement with single-unit recordings in monkeys (Killian et al., 2012), two recent studies observed grid-like activity in entorhinal BOLD signal representing visual space in a 2D stimulus array (**Figure 2C**; Julian et al., 2018; Nau et al., 2018), suggesting that allocentric spatial coordinates are extracted from such arrays and used to code spatial positions, even in the absence of navigation and explicit memory demands (Julian et al., 2018).

### Oscillatory Activity

**FIGURE 3:**
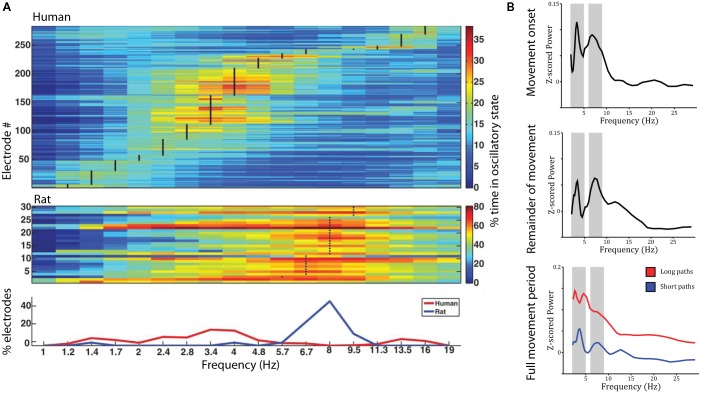
Theta oscillations during virtual navigation. **(A)** The predominant frequency of oscillations occurring during virtual navigation appears to be lower in humans than rats. A clear peak around 8 Hz is evident in rats, while human data shows a peak around 3–4 Hz. Adapted with permission (Watrous et al., 2013a) from Wiley Periodicals. **(B)** Theta oscillations are evident in the power spectrum during movement onset (*top*), the remainder of the movement period (*middle*), and the full movement period (*bottom*) in two distinct frequency bands around 3–4 and 8–9 Hz, at the edges of the conventional theta rhythm. Theta oscillations are of higher amplitude during long compared to short paths (*bottom*).

The studies reviewed above suggest that the place- and grid-cell network is largely conserved across species. Similar convergence across species has been observed with respect to neural oscillations. Most prominently, theta oscillations (4–8 Hz) have been observed in the rodent (Vanderwolf, 1969; Buzsáki et al., 1983) and human MTL during navigation compared to stillness (**Figure 3A**). Using intracranial electroencephalography (iEEG) in humans, theta has been observed in a radial arm maze (Bohbot et al., 2017), an open-field (**Figure 3B**; Bush et al., 2017) and a city environment (Ekstrom et al., 2005), as well as during real-world ambulatory movement (Aghajan et al., 2017; Bohbot et al., 2017). In addition, magnetoencephalography (MEG) studies have source localized theta activity to the MTL (Cornwell et al., 2008; Kaplan et al., 2012). Theta oscillations were observed likewise in the hippocampus and parahippocampal gyrus (Ekstrom et al., 2005; Cornwell et al., 2008) and co-occurred with an increase in hippocampal BOLD contrast (Kaplan et al., 2012; although other studies did not show a significant relation between hippocampal theta and BOLD: Ekstrom et al., 2009; Ekstrom, 2010). They were further indexed not only by increased power but also by examination of raw traces and an oscillation detection algorithm, which discriminates narrow band oscillations lasting several cycles from broad band and/or transient power increases (Ekstrom et al., 2005). Based on studies in rodents, most studies on human navigation *a priori* restricted their analyses to the theta band. Some studies have considered a wider spectrum of low-frequencies, and their findings suggest that the spatial navigation rhythm might be of lower frequency in humans than in rodents (**Figure 3A**). These studies observed a power spectral peak in the delta band around 2–3 Hz (Clemens et al., 2013; Watrous et al., 2013a; Miller et al., 2018) or both a low and a high frequency peak (Bush et al., 2017). While one study suggests that differences in frequency partly relate to virtual vs. real-word navigation (Bohbot et al., 2017), others have speculated that a shift toward lower frequencies relates to the larger size of the human hippocampus compared to that of the rodent (Jacobs, 2014).

Low frequency activity has been shown to be a function of movement speed (fast > slow; Watrous et al., 2011), navigational goal (fixed-location landmark > aimless navigation; Cornwell et al., 2008; Watrous et al., 2011), view (Watrous et al., 2011), path length (long > short, during movement onset and the remainder of the path; **Figure 3B**; Bush et al., 2017), and familiarity of the environment (familiar > novel; Kaplan et al., 2012). These findings shed some light on the role of low frequency oscillations during navigation. Specifically, two (not mutually exclusive) hypotheses have been discussed: Low frequency oscillations might coordinate sensory and motor areas during navigation (Bland and Oddie, 2001; Caplan et al., 2003) or might play a role for spatial coding and memory (Cornwell et al., 2008; Watrous et al., 2011). Modulations by movement speed can be easily explained by a sensorimotor integration account (Bland and Oddie, 2001). Modulations by view, however, were observed as stronger theta power during viewing of non-goal buildings compared to goal buildings or a relatively uniform background (Watrous et al., 2011). These results are difficult to reconcile with a pure sensorimotor account, as they seem to indicate that theta activity specifically increases when viewing landmarks, which can be used to plan routes to the goal location, i.e., when encoding and retrieval of spatial information is required. Similarly, enhanced theta power during goal-directed navigation compared to aimless navigation suggests a role for theta oscillations in spatial memory retrieval and route planning. The same is true for higher theta power during movement onset for long compared to short paths (which require retrieval of more spatial information and planning of a longer route). Finally, higher theta power in familiar environments may relate to the fact that more stored spatial information is available that can be retrieved to guide movement. Taken together, these results suggest that theta oscillations are involved not only in low-level sensory and motor processes but also in the encoding and retrieval of spatial information.

Further support for the spatial memory hypothesis has been obtained from both rodent and human studies. In rodents, it has been shown that theta oscillations orchestrate the firing of place-responsive cells. Specifically, a place cell fires at progressively earlier phases of the theta cycle while a rat traverses its place field (O’Keefe and Recce, 1993), a phenomenon that has been termed phase precession and implicates theta oscillations in representing allocentric spatial location. In humans, hippocampal theta-power has been associated with navigation performance across subjects (Cornwell et al., 2008) and with pre-navigation planning across trials: Upon instruction to find the location of a previously presented object, theta power was found to be higher for subsequently accurately vs. inaccurately placed objects (Kaplan et al., 2012). Miller et al. (2018) have associated slow theta power with successful encoding of object-location pairs. In their task, subjects navigated a virtual arena to reveal objects. During retrieval, they were cued with objects and had to recall the associated location. Subsequently successfully placed objects were associated with higher slow theta power during encoding. Finally, one study used a virtual environment equipped with multiple teleporters to decouple traveled distance from sensory input and motor output during navigation. Here, delta–theta oscillations were predictive of the spatial distance traveled during teleportation (i.e., in the absence of sensory-motor demands) while controlling for the time being teleported (Qasim and Jacobs, 2016; Vass et al., 2016).

While most studies *a priori* focused on low frequency oscillations, few studies have analyzed modulations in higher frequencies. These studies have found navigation-related increases in hippocampal and parahippocampal alpha (∼9–14 Hz), beta (∼15–30 Hz), and gamma (∼31–55 Hz) power (Ekstrom et al., 2005; Jacobs et al., 2009; Watrous et al., 2011). An increase in power, however, does not necessarily index underlying oscillations but may also be caused by transient, non-oscillatory amplitude changes (Whitten et al., 2011). While the presence of narrowband oscillations in the low frequency range has been established by multiple studies using oscillation detection algorithms (Caplan et al., 2003; Ekstrom et al., 2005; Watrous et al., 2011), it remains largely unclear to what extent effects in higher frequencies are due to broadband shifts in spectral power versus narrowband oscillations. Likewise, the functional role of medial temporal high frequency effects in navigation remains a subject for future study.

## Spatial Representations in a Brain-Wide Network

**FIGURE 4:**
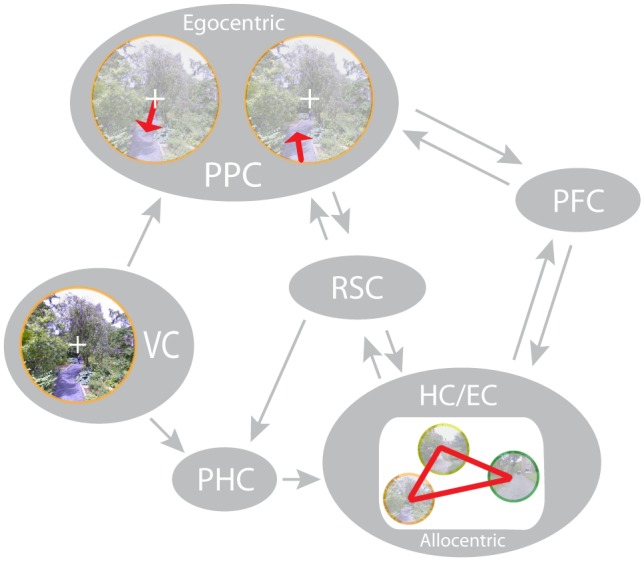
Key regions involved in spatial memory and navigation. The PPC, situated along the dorsal visual stream, extracts spatial coordinates from visual input and translates between different egocentric reference frames (e.g., retinotopic or head-centered). These can be used to track motion and plan movements in coordination with the PFC. The PHC receives input not only via the ventral visual pathway but also from the dorsal pathway via the RSC. It projects to the HC via the EC, where allocentric coding pre-dominates. The RSC might translate between parietal egocentric and medial temporal allocentric representations. Prefrontal interactions with HC and surrounding MTL may facilitate goal-directed navigation. EC, entorhinal cortex; HC, hippocampus; MTL, medial temporal lobel; PHC, parahippocampal cortex; PPC, posterior parietal cortex; RSC, retrosplenial cortex; VC, visual cortex.

Although place- and grid-like activity has mainly been associated with the hippocampus and entorhinal cortex, respectively, studies have also observed such signals outside the MTL. Specifically, cells in PFC showed place-selectivity (Ekstrom et al., 2003) and cells in cingulate cortex showed both, place and grid-like firing (Jacobs et al., 2013; **Figure 1E**). Using fMRI with whole brain coverage, wide-spread grid-like activity was observed in medial prefrontal, parietal, and lateral temporal cortices (Doeller et al., 2010). Similarly, navigation-related low- and high-frequency oscillations are prevalent not only in the MTL but also in frontal (Caplan et al., 2001, 2003; Ekstrom et al., 2005; Jacobs et al., 2009; Kaplan et al., 2012), lateral temporal (Kahana et al., 1999; Caplan et al., 2001; de Araujo et al., 2002; Ekstrom et al., 2005; Jacobs et al., 2009), parietal and occipital cortex (Jacobs et al., 2009). Theta oscillations are correlated between hippocampus and neocortex as well as between different cortical regions (Ekstrom et al., 2005). Further, low-frequency phase consistency between the parahippocampal gyrus and sub-regions in frontal and parietal cortex has been implicated in retrieval of spatial information (Watrous et al., 2013b). Taken together, these results demonstrate that spatial representations are not strictly confined to the MTL and that interactions between medial temporal and distant cortical brain regions support the encoding and retrieval of spatial relations to successfully orient oneself in and navigate the surrounding environment (**Figure 4**; Ekstrom et al., 2017).

Interactions between the MTL and posterior brain regions may underlie encoding of spatial relations to build a cognitive map and to relate current perceptual input to information stored in that map. Two separate neural pathways have been described in primate vision (Goodale and Milner, 1992), connecting visual with temporal areas (ventral “what” pathway) for object recognition and visual with parietal areas (dorsal “how” or “where” pathway) for object localization, respectively. While the latter pathway has traditionally been thought to mainly project to motor areas in dorsal frontal cortex for spatially accurate movement planning, anatomical, and functional evidence suggests that egocentric spatial maps in parietal cortex also provide strong inputs to MTL structures (especially to the hippocampus and parahippocampal cortex), both directly, and via the posterior cingulate and retrosplenial cortices (RSC) (Kravitz et al., 2011). This input carries information on egocentric distances and self-motion extracted from binocular disparity (Georgieva et al., 2009) and optic flow (Siegel and Read, 1997; Wall and Smith, 2008). Accordingly, the PPC is more active during virtual navigation than during viewing of static scenes as indexed by regional cerebral blood flow measured with PET (Maguire et al., 1998) and during active navigation compared to passive traversal of a repeated corridor as indexed by fMRI BOLD (Wolbers and Buechel, 2005). Furthermore, calculation of an egocentric versus an allocentric homing vector is associated with a more parietal versus temporal source activity distribution as obtained from scalp EEG (Gramann et al., 2006).

As a relay between PPC and MTL, the RSC contains head direction cells in rodents (Chen et al., 1994) and is strongly active during viewing familiar scenes, during identification of the location associated with a known scene, and during imagined navigation, as shown with fMRI BOLD (Ino et al., 2002; Epstein et al., 2007a,b; Epstein and Higgins, 2007). Based on these findings, it has been speculated that the RSC translates between parietal egocentric and medial temporal allocentric reference frames (Burgess, 2006; Epstein, 2008). The precise neurophysiological interactions between human PPC, RSC, and MTL, however, remain to be investigated.

Interactions between the MTL and frontal brain regions, in turn, may underlie retrieval of spatial information in the course of action planning with respect to current goals. Neuroimaging has shown that the PFC is more active during successful compared with unsuccessful navigation, during active compared with guided navigation and when an unexpected detour is required (Maguire et al., 1998; for a review see Spiers and Gilbert, 2015). Further, using fMRI and a model-based learning algorithm, Simon and Daw (2011) showed that the value associated with a chosen path was predictive of prefrontal BOLD signal. These studies specifically implicate the PFC in prospective evaluation and selection of possible routes. The PFC might thereby access stored information on goal location and available paths held in the MTL. Rodent studies show that hippocampal place cells exhibit spiking outside of their place fields (i.e., non-local place representations) at decision points (Johnson and Redish, 2007). In humans, single units in both frontal and medial temporal lobe represent current goal locations (Ekstrom et al., 2003), implicating coordinated activity between MTL and PFC in planning goal-directed behavior. More direct evidence for interactions between MTL and PFC during route planning has been provided with fMRI: Brown et al. (2016) showed that univariate activation in the frontopolar cortex, as well as the strength of orbitofrontal goal representations covaries with the strength of hippocampal goal representations. Coordination between these brain regions may rely on theta synchrony: During a cue period specifying the goal of a subsequent navigation period, prefrontal theta oscillations have been shown to exhibit phase-locking (i.e., a consistent phase-difference) to the hippocampal theta rhythm (Kaplan et al., 2014).

## Cognitive Mapping and Complementary Learning Mechanisms in Human Spatial Navigation

Although electrophysiological evidence aligns with the concept of an allocentric spatial map, behavioral work supports the flexible use of multiple cognitive representations during navigation. In the present section, we highlight some of the central ideas related to this multiple-representations perspective (a more comprehensive treatment can be found in, e.g., Burgess, 2006; Khamassi and Humphries, 2012; Ekstrom et al., 2014; Wolbers and Wiener, 2014; Filimon, 2015).

Evidence consistent with the assumption of an allocentric spatial map has been provided by Manning et al. (2014) using a computational modeling approach. Their model accounts for navigational behavior under the assumption that subjects encode and retrieve associations between landmarks and their perceived location within an allocentric spatial map of the environment. Memory for these associations, which form and decay during navigation, is used to determine an optimal path toward a target location within the environment. Their model could accurately account for subjects’ spatial knowledge expressed in excess path length and a pointing task. Moreover, pointing performance was higher while subjects’ view was aligned with a salient axis of the environment (i.e., north–south or east–west), as compared with when it was unaligned. This alignment effect (which is not to be confused with the alignment of movement and grid direction discussed in section “Spatially Selective Single Cells – Observed via Invasive Recordings or Inferred from Population Activity”) suggests that subjects rely (at least to some degree) on an allocentric spatial reference frame centered on these axes: The mental rotation required when pointing from an unaligned view introduces additional error compared to aligned pointing. Similar alignment effects were observed in other studies (McNamara et al., 2003; Brunyé et al., 2015). McNamara et al. (2003) further showed that the orientation and origin of the allocentric coordinate system used depends on egocentric heading during exploration. Simon and Daw (2011) compared a model-based algorithm to an opposing cue-response learning strategy. Here, values associated with responses to specific landmarks are learned based on a temporal difference reinforcement learning (TD-RL) algorithm that is blind to the global spatial structure of the environment. A direct comparison between these two revealed an average Bayes factor of 17, providing strong evidence in favor of model-based planning. Finally, Chen et al. (2015) examined failures in distance estimation following rescaling of a known virtual arena. They show that errors in path integration are predicted by the way grid cells rescale their firing fields upon such changes in environmental shape in rodents (Barry et al., 2007), providing a link between grid cell activity and human navigation.

A different set of studies, in contrast, suggests that human subjects acquire local spatial knowledge, but do not integrate that knowledge into a coherent global spatial map. In one study, Warren et al. (2017), created a virtual environment that contained “invisible wormholes,” which teleported subjects between pre-determined locations without any perceptual cue. The only way for subjects to detect the teleportation would therefore be an awareness for the geometric inconsistencies caused by the presence or absence of teleportation on different routes to a target location. Although subjects were able to navigate successfully to two locations A and B from a third location C (two paths not containing a wormhole), when being asked to walk from A to B (a path containing a wormhole during learning), subjects showed a strong bias toward the “experienced wormhole location” of the target location. None of the subjects, however, reported any experienced inconsistencies, suggesting that no global metric map of the environment was formed. Another study examined navigation in an environment consisting of several enclosed local spaces (i.e., buildings) and came to a similar conclusion: Here, subjects often failed to navigate to a correct global location while being able to locate an object correctly in local dimensions (i.e., they navigate to the correct location in the wrong building) (Marchette et al., 2017). Finally, two studies examined object-location memory within and across spatial boundaries. They showed that pointing across spatial boundaries (rooms or neighborhoods) is slower and less accurate compared to within-boundary pointing (Han and Becker, 2014; Meilinger et al., 2016). These findings highlight the impact of spatial scale on memory and navigation (Wolbers and Wiener, 2014) and suggest that subjects’ spatial knowledge contains local geometric information which is not always integrated into a coherent Euclidean map.

Navigation in large-scale complex environmental spaces may depend on multiple learning strategies. A common taxonomy of navigation distinguishes a model-based allocentric place strategy from a model-free egocentric cue-response strategy. However, one can imagine situations in which the representational reference system (egocentric cue/allocentric place) is independent of the type of learning (model-free/model-based). Specifically, allocentric place representations can cue a habitual response (Foster et al., 2000) and associations between landmarks can be learned in a model-based rather than a model-free fashion (Khamassi and Humphries, 2012), allowing for graph-like knowledge of spatial relations (Chrastil, 2013; Warren et al., 2017). In addition, learning might take place at an intermediate level of flexibility and computational expense, using what has been introduced as the successor representation (Dayan, 1993; Momennejad et al., 2017). Instead of directly caching action-values (i.e., model free) or storing a full map of all possible state transitions that is combined with a value function during decision making (i.e., model based), agents might cache predictions about future states (i.e., how often each successor state will be visited in the future), which they can similarly combine with a value function during decision making. Caching the number of expected future visits is less expensive than storing a complete map of the world (i.e., model-based), but more flexible than model-free learning when changes to the reward structure (e.g., a change in goal) occur, since value function and spatial knowledge are stored separately. The successor representation has recently been used to explain the firing of place responsive cells (Stachenfeld et al., 2017), but has not yet been directly linked to human navigational behavior. Formal models that account for such alternative learning and decision mechanisms might help to develop a better understanding of navigation in large environmental spaces and, ultimately, its neural underpinnings.

## Functional Overlap in the MTL: A Common Map for Physical and Conceptual Space?

**FIGURE 5:**
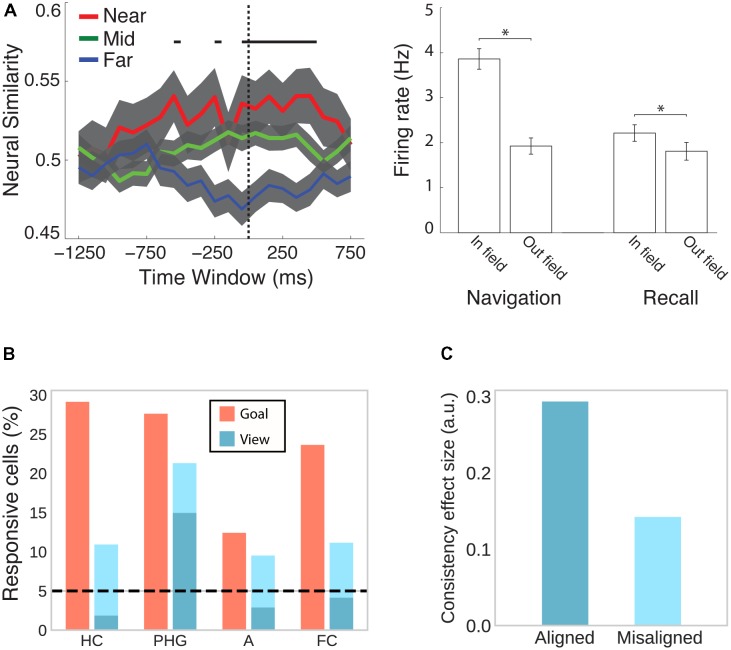
Cells in the human MTL code non-spatial features as well as spatial features outside their respective spatial context. **(A)** While viewing a black screen, the activity of place cells is reinstated when subjects recall words that were encoded inside the cells’ place field in a virtual city environment. This effect is evident at the population (*left*) and individual cell (*right*) level. Adapted with permission (Miller et al., 2013) from Science. **(B)** Goal- and view- responsive cells in the hippocampus (HC), parahippocampal gyrus (PHG), amygdala (A), and frontal cortex (FC). *Dark turquoise bars* indicate responsiveness to stores and *light turquoise bars* indicate responsiveness to both stores and passengers. Adapted with permission (Ekstrom et al., 2003) from Springer Nature. **(C)** Evidence from fMRI suggests that cells in the entorhinal cortex show grid-like activity that codes position in a conceptual space representing visual features of birds (neck length and leg length). BOLD contrast was higher for viewing or imagining morphing trajectories that were aligned with the common grid orientation as compared with misaligned trajectories. Data from Constantinescu et al. (2016).

The MTL is not only the major focus of electrophysiological studies investigating the neural signature of spatial memory, but, ever since the hallmark findings on patient H.M. (Scoville and Milner, 1957; Milner et al., 1968), it has also emerged in human neuroimaging as a central brain region for declarative memory more generally (Mayes et al., 2007; Battaglia et al., 2011). A study combining spatial navigation with episodic free recall has shown that place-responsive cells in the human MTL reinstate their activity during recall of items that were encoded in the cell’s place field (**Figure 5A**; Miller et al., 2013), suggesting that place cells do not only code instantaneous spatial position but also represent a spatial code for remembering past episodes.

Besides coding instantaneous as well as remembered spatial locations, hippocampal and entorhinal cells have been shown to code a variety of other features. In rodents, hippocampal and entorhinal cells have been associated with the coding of elapsed time (Pastalkova et al., 2008; MacDonald et al., 2011; Kraus et al., 2013; for a review see Eichenbaum, 2014), behavior (i.e., approach), stimulus quality (i.e., odor identity), and task characteristics (i.e., match vs. non-match trials) (Wood et al., 1999). In humans, cells in the MTL are responsive to view of or search for a specific landmark irrespective of the position of the observer (**Figure 5B**; Ekstrom et al., 2003). Moreover, a subset of place-responsive cells has been shown to remap (i.e., change their place field) upon a change in goal location (Ekstrom et al., 2003), suggesting that the same cells are responsive to spatial and non-spatial features during navigation. Outside of navigation, human MTL cells are sensitive to the identity of individuals, landmarks or objects irrespective of the type of presentation (i.e., pencil sketches, photographs, letter strings, etc.) (Quiroga et al., 2005). Using fMRI, Constantinescu et al. (2016) further observed grid-like activity coding imagined trajectories in conceptual space (**Figure 5C**). Subjects first learned a conceptual two-dimensional space of visual bird features (i.e., neck and leg length) and subsequently viewed and imagined trajectories in this space (i.e., birds morphing along a given neck:leg length ratio). Grid-like modulation of BOLD was observed in entorhinal cortex, posterior cingulate and retrosplenial cortices, PPC, temporo-parietal junction, and PFC. Collectively, these studies implicate the spatial memory network described above more broadly in coding associations between different kinds of features in the service of perception, memory, and prospective planning (Eichenbaum et al., 1999; Buzsáki, 2005; Buzsáki and Moser, 2013; Eichenbaum and Cohen, 2014).

One way to approach this high degree of functional overlap in the MTL is to identify functional subdivisions and link them to the encoding and retrieval of different classes of stimulus features. In the episodic memory literature, a popular model of MTL function assigns item processing to the perirhinal and lateral entorhinal cortex, (spatial) context processing to the parahippocampal and medial entorhinal cortex, and item-in-context processing to the hippocampus (Eichenbaum et al., 2012; see Buffalo et al., 2006 and Wixted and Squire, 2011 for evidence that perirhinal cortex also processes spatial information). This model has received support from studies on functional connectivity, which have embedded these structures into a broader anterior temporal (item) and posterior medial (context) network (Ranganath and Ritchey, 2012; Ritchey et al., 2015). The distinction between item processing in lateral and spatial context processing in medial entorhinal cortex in this model is in line with the finding that grid cells in rats are localized to the medial (rather than lateral) entorhinal cortex (Hafting et al., 2005; Moser et al., 2008). Studies on grid cells in humans, however, have so far been using a spatial resolution too coarse to identify such functional subdivisions. Ultra-high field MRI at 7T has recently been used to successfully differentiate an antero-lateral and a posterior-medial sub-region of the human entorhinal cortex based on resting state connectivity, as well as differential processing of objects and scenes (Maass et al., 2015; Schröder et al., 2015; for a review see Schultz et al., 2015), suggesting that future studies should in principle be able to study differential engagement of these entorhinal regions in coding spatial vs. non-spatial features.

**FIGURE 6:**
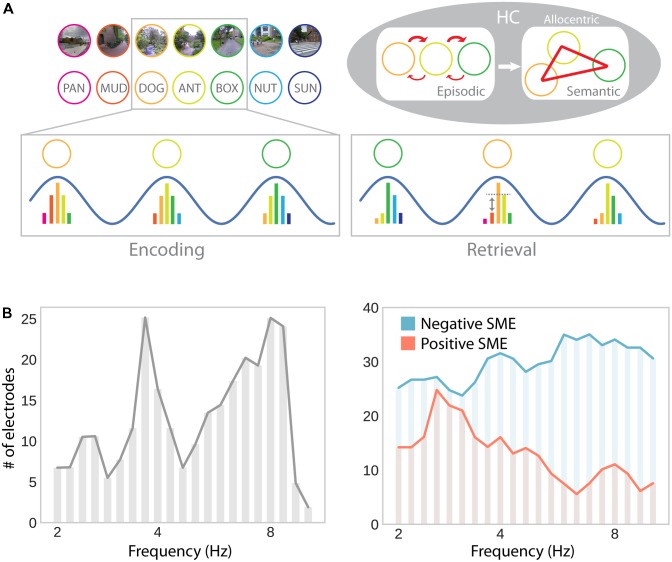
Theta oscillations in episodic memory formation and retrieval. **(A)** Model suggesting a common role for hippocampal theta oscillations and phase precession in memory for spatial and non-spatial sequences (e.g., during navigation or a list-learning task; Buzsáki, 2005). Successive firing of hippocampal cells within a theta cycle establishes temporal associations between both place and item representations through synaptic plasticity, which favors associations in the forward direction. Multiple encounters of the same items/places in different serial order establishes semantic/allocentric representations of conceptual and physical space. In part adapted with permission (Buzsáki, 2005) from Wiley Periodicals. **(B)** Hippocampal electrodes showing theta oscillations around 3–4 and 8–9 Hz, analogously to theta effects during navigation (see **Figure 3B**; *left*), as well as positive and negative subsequent memory effects (SME; *right*). Positive SMEs predominate around 3–4 Hz. Adapted with permission (Lega et al., 2012) from Wiley Periodicals.

An alternative (and complementary) approach is to develop a unifying framework that supports spatial and non-spatial memory functions with the same mechanisms. Buzsáki (2005) suggested that associations between spatial and non-spatial information are established through theta oscillations in the hippocampus, giving rise to episodic memory, unidirectional place cells, semantic memories, and omnidirectional place cells. Specifically, precession of hippocampal cells to the theta rhythm (i.e., progressively earlier spiking as the place field is traversed) results in multiple cells firing within a single theta cycle. These cells represent successively visited places or successively presented items (e.g., in a non-spatial recall task; **Figure 6A**). Consequently, the synaptic connections between these cells get strengthened by spike timing-dependent plasticity, which favors associations in the forward direction. This time compression mechanism explains unidirectional place cells in one-dimensional navigation: As connections are specifically strengthened in the forward direction, traversal in the same direction reinstates firing of the same sequence of cells, whereas traversal in the opposite direction does not. Similarly, it explains two hallmark findings of episodic free recall: Temporal contiguity (i.e., stronger associations between items that were encoded in temporal proximity) and temporal asymmetry (i.e., stronger associations in the forward than the backward direction) (Kahana, 1996). Time-independent associations, which give rise to omnidirectional place cells and semantic memory, in this framework, are formed from multiple overlapping traversals of a given location, as well as multiple overlapping encounters of a given item (Buzsáki, 2005).

While this idea elegantly unites place cells, theta oscillations, spatial, episodic and semantic memory, it has yet to be verified experimentally. If theta oscillations provide a time compression mechanism that can act independently of spatial navigation and establishes temporal associations between successively experienced items, evidence consistent with this idea should be observable in electrophysiological studies of human recall and recognition. Although a large number of studies highlight a role for low-frequency oscillations in episodic memory encoding, they provide mixed results of increases (Guderian et al., 2009; Hanslmayr et al., 2011; Lega et al., 2012; Lin et al., 2017) and decreases (Sederberg et al., 2007; Lega et al., 2012; Burke et al., 2013; Lin et al., 2017; Solomon et al., 2018) associated with successful encoding in the MTL, as well as in cortical brain regions or at the scalp (increases: Klimesch et al., 1996; Osipova et al., 2006; Sederberg et al., 2007; Khader et al., 2010; Burke et al., 2013; decreases: Sederberg et al., 2007; Guderian et al., 2009; Burke et al., 2013). It is noteworthy that several of these studies simultaneously report both increases and decreases which are separated in time and/or precise localization (Sederberg et al., 2007; Guderian et al., 2009; Lega et al., 2012; Burke et al., 2013, 2014). Similarly, when looking at theta phase synchrony, rather than theta power, studies have observed increases (Burke et al., 2013; Solomon et al., 2017) as well as decreases (Burke et al., 2013) in brain-wide theta synchrony associated with successful memory formation.

The results reported by Lega et al. (2012) seem to be particularly interesting with respect to navigation-related theta effects: They show two distinct peaks in low (∼3–4 Hz) and high (∼8 Hz) frequency theta oscillations. Furthermore, subsets of hippocampal depth electrodes show increases and decreases in theta oscillations as a function of subsequent memory. The co-occurrence of these effects explains why both increases and decreases may be observed in average theta power depending on sampling and measurement procedures. Furthermore, positive and negative subsequent memory effects seem to be differentially linked to slow (∼3–4 Hz) and fast (∼8 Hz) theta oscillations, respectively (**Figure 6B**). These results thereby partially resolve the discrepancies between the navigation and episodic memory literature in suggesting that a localized slow hippocampal delta–theta rhythm is involved in successful encoding and navigation (see section “Oscillatory Activity”), whereas a faster theta rhythm is detrimental for episodic encoding (see Ekstrom and Watrous, 2014 and Watrous et al., 2013b for an alternative spectral fingerprinting account of slow and fast theta in spatial and episodic memory). Based on the idea that theta oscillations specifically facilitate inter-item or item-context associations (Buzsáki, 2005), one could further argue that the more informative contrast in this regard should involve some form of successful context encoding (rather than just successful item encoding). However, few studies report such a contrast and, again, provide mixed results: While Staudigl and Hanslmayr (2013) observe a theta (∼4 Hz) increase during successful item-in-context encoding, Long and Kahana (2017) observe no significant difference in theta power for items subsequently retrieved with or without temporal context information. Overall, one can say that the evidence for theta power increases associated with successful episodic encoding are far less robust and effects are less distributed across the brain, compared to findings obtained during navigation.

We have outlined above that theta oscillations play a role not only in encoding but also retrieval of spatial information from memory and imagined navigation in a familiar environment. Theta effects during episodic retrieval seem generally more coherent than encoding effects. Theta increases have been shown to precede spontaneous recall (Burke et al., 2014) and to differentiate successful recollection of contextual information from item recognition (Guderian and Düzel, 2005; Herweg et al., 2016). While MEG effects were source localized to the MTL (Guderian and Düzel, 2005), intracranial effects were localized mainly to the anterior temporal cortex (Burke et al., 2014). It remains an open question whether this difference is due to imprecise source localization or differences in the cognitive demands posed by the retrieval tasks (i.e., free recall vs. source memory judgment). Evidence for the role of hippocampal theta oscillations during memory retrieval has also been provided by studies investigating theta mediated synchrony. One study linked theta power increases in scalp EEG to hippocampal connectivity in BOLD data (Herweg et al., 2016). Specifically, a psychophysiological interaction analysis revealed connectivity between hippocampus and other brain regions in the core memory network to be positively associated with theta power. Another study observed increased brain-wide theta phase synchronization (involving the hippocampus) as measured with iEEG during successful episodic recall (Solomon et al., 2017). These findings highlight the role of theta oscillation during retrieval of non-spatial information, and thereby parallel findings on spatial memory.

A unifying account of MTL function should account for the role of theta during both encoding and retrieval of spatial and non-spatial memories. One possibility is that theta oscillations during retrieval organize spike timing (as they do during encoding) to represent temporal context and remembered or imagined serial order information (**Figure 6A**). Given the wealth of evidence demonstrating the importance of retrieval for learning (Karpicke and Roediger, 2008; Roediger and Karpicke, 2006a,b), it is likely that theta’s role in spike-timing dependent plasticity ought to operate during retrieval as well as encoding. Specifically, theta oscillations during retrieval might facilitate re-encoding of temporal associations between retrieved/imagined items/places. These assumptions can be tested by linking theta oscillations during retrieval to immediate and future associative memory strength.

## Concluding Remarks

We have reviewed converging evidence that the human MTL is equipped with a population of place and grid cells that provides an allocentric spatial map of the environment, similar to that observed in the rodent brain (see section “Spatially Selective Single Cells – Observed via Invasive Recordings or Inferred From Population Activity”). Furthermore, spatial coding in the human MTL seems to be supported by oscillatory activity in the theta frequency range (although this may be lower in humans than rodents; see section “Oscillatory Activity”). Recent findings strongly suggest that theta oscillations are not only involved in sensorimotor integration, as has previously been argued, but instead directly relate to the encoding and retrieval of spatial information. Their relation to spiking activity of place-responsive cells remains less clear and, hence, a subject for future study.

Despite the evidence for an allocentric spatial map in the human MTL, we have pointed out that the MTL does not work in isolation (see section “Spatial Representations in a Brain-Wide Network”). Connections with parietal brain regions provide input to the MTL that carries egocentric spatial information and prefrontal brain regions make use of the allocentric spatial map in the process of prospective thinking and action selection. Moreover, an allocentric spatial map alone does not fully account for the affordances of human spatial navigation in environmental spaces and, presumably, real-world settings (see section “Cognitive Mapping and Complementary Learning Mechanisms in Human Spatial Navigation”). The interactions of different spatial learning mechanisms and navigation strategies are currently not well understood, both at the behavioral and neurophysiological level. Clearer taxonomies along with stronger consideration of the cognitive demands posed by different environments will help to develop and refine formal models of navigational behavior that can be linked to neurophysiological phenomena.

Finally, a parallel line of research has implicated the MTL in declarative memory formation and retrieval (see section “Functional Overlap in the MTL: A Common Map for Physical and Conceptual Space?”). It remains to be determined to what degree this functional overlap can be either resolved by identifying sub-regions and networks in the MTL that preferentially process spatial vs. non-spatial information or explained with a common framework that integrates spatial and declarative memory. We believe that a critical step in developing and refining such a unified theory of MTL function will be to specifically link task-related phenomena at multiple temporal and spatial scales. Understanding how place- (and concept-) responsive single cell activity relates to ongoing theta oscillations during both the encoding and retrieval of spatial and non-spatial associations will significantly contribute to a more mechanistic understanding of memory processes in the MTL.

## Outstanding Questions

(1) What is the relation between spiking activity of place-responsive cells and the hippocampal theta rhythm in humans, and how is information coded in spiking-phase relations? Although Jacobs et al. (2007) found general evidence for phase-locking of single neurons to theta oscillations, this relation has not been specifically linked to spatially responsive neurons in the MTL. Furthermore, rodent work demonstrates that place (O’Keefe and Recce, 1993) and grid (Hafting et al., 2008) cells show phase precession to the hippocampal theta rhythm while rats traverse a place field. It remains unknown whether human place-responsive cells demonstrate similar spike-phase relations.
(2) Whereas studies of rodent spatial coding often use small-scale spaces, studies in humans often use large-scale virtual environments with constrained paths (Wolbers and Wiener, 2014). How do these different paradigms affect coding of space and navigational strategies? Can a closer examination of navigational paradigms reconcile some of the inconsistencies between electrophysiological findings supporting and behavioral findings contradicting a Euclidean metric map (e.g., Meilinger et al., 2016; Marchette et al., 2017; Warren et al., 2017)?
(3) Both rodents and humans preferentially navigate on flat surfaces and study of their spatial navigation system has consequently focused on two dimensional spatial representations. Place cells in the bat hippocampus, in turn, have been shown to exhibit isometric three dimensional place fields that are tuned to the affordances of volumetric navigation (Yartsev and Ulanovsky, 2013; Finkelstein et al., 2016). How do humans code spatial locations in multilayered navigation, on elevated surfaces, or during volumetric navigation (e.g., while diving or in an aircraft)? Preliminary evidence suggests speed but not accuracy costs associated with spatial memory in the vertical compared to the horizontal dimension of a 3D environment (Kim et al., 2017). Moreover, partially overlapping brain regions seem to encode vertical and horizontal space (Kim et al., 2017; Kim and Maguire, 2018). It remains to be shown, however, how the network of human place and grid cells codes 3D space. If the spatial representation system is not only used to code physical, but also conceptual spaces, how does the dimensionality of preferred navigation affect the representation of conceptual space (which is not always two dimensional)?
(4) Some studies suggest that the navigation-related theta rhythm is slower in humans than in rodents (Jacobs, 2014) or that oscillations occur in separate low- and high- frequency theta bands (Bush et al., 2017). Studies in the episodic memory domain provide mixed evidence on the direction of theta effects during successful encoding. Results reported by Lega et al. (2012) suggest that oscillations in the low-theta band facilitate encoding, while oscillations in the high-theta band are detrimental for performance, raising a potential way to reconcile the findings. Are there indeed two separate theta rhythms? What is their differential role in navigation and episodic memory?
(5) It has been shown that spiking activity of place cells is reinstated when subjects remember an item that was encoded within a cell’s place field (Miller et al., 2013), implicating place cells in the retrieval of item-in-spatial-context information during episodic recall. Do grid cells show similar reinstatement effects during episodic memory retrieval or is their role more specifically tied to spatial navigation and path integration?
(6) Buzsáki (2005) has proposed an elegant theory explaining the emergence of spatial and semantic maps from episodic experience. Is there direct evidence in favor of the idea that theta oscillations provide a time compression mechanism that establishes associations between successively experienced items in an episodic memory task in humans?

## Author Contributions

NH and MK performed the literature search and discussed the results. NH drafted the manuscript. NH and MK revised the manuscript, approved the final version, and agreed to be accountable for all aspects of the work.

## Conflict of Interest Statement

The authors declare that the research was conducted in the absence of any commercial or financial relationships that could be construed as a potential conflict of interest. The reviewer QH and handling Editor declared their shared affiliation.
